# Evolutionary consequences of feedbacks between within-host competition and disease control

**DOI:** 10.1093/emph/eoaa004

**Published:** 2020-02-04

**Authors:** Megan A Greischar, Helen K Alexander, Farrah Bashey, Ana I Bento, Amrita Bhattacharya, Mary Bushman, Lauren M Childs, David R Daversa, Troy Day, Christina L Faust, Molly E Gallagher, Sylvain Gandon, Caroline K Glidden, Fletcher W Halliday, Kathryn A Hanley, Tsukushi Kamiya, Andrew F Read, Philipp Schwabl, Amy R Sweeny, Ann T Tate, Robin N Thompson, Nina Wale, Helen J Wearing, Pamela J Yeh, Nicole Mideo

**Affiliations:** e1 Department of Ecology & Evolutionary Biology, University of Toronto, 25 Willcocks St., Toronto, ON M5S 3B2, Canada; e2 Department of Zoology, University of Oxford, Zoology Research and Administration Building, 11a Mansfield Road, Oxford OX1 3SZ, UK; e3 Department of Biology, Indiana University, 1001 E. 3rd St., Bloomington, IN 47405, USA; e4 Odum School of Ecology and the Center for the Ecology of Infectious Diseases, University of Georgia, 140 E Green St., Athens, GA 30602, USA; e5 Department of Biology, Emory University, Atlanta, GA 30322, USA; e6 Department of Mathematics, McBryde Hall, Virginia Tech, Blacksburg, VA 24061, USA; e7 Institute of Integrative Biology, University of Liverpool, Liverpool, L69 3BX, UK; e8 Institute of Zoology, Zoological Society of London, Regent’s Park, NW1 4RY, UK; e9 Departments of Mathematics & Biology, Jeffery Hall, Queen’s University, Kingston, ON K7L 3N6, Canada; e10 Institute of Biodiversity, Animal Health & Comparative Medicine, University of Glasgow, Glasgow G12 8QQ, UK; e11 CEFE UMR 5175, CNRS - Université de Montpellier, Université Paul-Valéry Montpellier, EPHE, 1919, Route de Mende, 34293 Montpellier Cedex 5, France; e12 Department of Integrative Biology, Oregon State University, 3029 Cordley Hall Corvallis, OR 97331, USA; e13 Department of Evolutionary Biology and Environmental Studies, University of Zürich, Zürich, 8057, Switzerland; e14 Department of Biology, New Mexico State University, Foster Hall, Las Cruces, NM 88003, USA; e15 Center for Infectious Disease Dynamics, Huck Institutes for the Life Sciences; Departments of Biology and Entomology, Pennsylvania State University, University Park, PA 16802, USA; e16 Department of Biological Sciences, Vanderbilt University, Nashville, TN 37235, USA; e17 Mathematical Institute, University of Oxford, Woodstock Road, Oxford OX2 6GG, UK; e18 Christ Church, University of Oxford, St Aldates, Oxford OX1 1DP, UK; e19 Department of Ecology & Evolutionary Biology, University of Michigan, 1105 North University Ave, Biological Sciences Building, Ann Arbor, MI 48109, USA; e20 Departments of Biology and Mathematics & Statistics, The University of New Mexico, Albuquerque, NM 87131, USA; e21 Department of Ecology & Evolutionary Biology, University of California, Los Angeles, 621 Charles E Young Dr South, Los Angeles, CA 90095, USA

## Abstract

Lay Summary: Competition often occurs among diverse parasites within a single host, but control efforts could change its strength. We examined how the interplay between competition and control could shape the evolution of parasite traits like drug resistance and disease severity.

Parasites often share their hosts with other parasites, meaning that hosts infected by a single, homogenous parasite population represent a rare exception. More commonly, hosts are infected by a variety of pathogenic organisms—any of which we refer to as ‘parasites’—that may themselves comprise multiple genetic variants (‘strains’). Such diversity within the host can lead to competition for resources or for respite from the shared threat of host immune defenses. Within-host competition has long been a focus of theoretical and experimental research in evolutionary ecology, and it is now widely appreciated that competition can alter the evolutionary trajectories of key parasite traits like virulence (reviewed in [1]). Equally widespread is the recognition that medical and public health interventions also drive the evolution of parasite traits; drug resistance, for example, has made its way to the forefront of our common conscience and our newsfeeds. Yet the interactions between these two sources of selection pressure are underexplored despite their inextricable linkage: by limiting transmission between hosts and/or inhibiting replication within hosts, control efforts—when successful—are likely to reduce the frequency and intensity of within-host competition. The evolutionary consequences of such interactions for parasite traits are as yet unmapped, and the implications for host health and disease control remain uncertain.

Seeking to fill this gap and elucidate common principles—or lack thereof—shaping parasite evolution in the presence of competition and disease control, we recently held an interdisciplinary workshop on this topic at Princeton University. The workshop was organized by Nicole Mideo and Megan A. Greischar as part of the NSF-funded Infectious Disease Evolution Across Scales (IDEAS) Research Coordination Network, with a combination of invited speakers and participants selected based on blinded review of applications (see author list). Here, we use the main discussion points of the workshop as a guide to define the level of understanding required to anticipate the evolutionary impact of feedbacks among within-host interactions, epidemiological processes and disease control, and we identify key open challenges for generating this understanding.

## OUTSTANDING QUESTIONS IN EVOLUTION, COMPETITION AND DISEASE CONTROL

### When do single versus multiple infections yield distinct health outcomes?

In evolutionary theory, infections are often categorized into two types: single or multiple infections, with the latter implying two or more different parasite strains (or species). This simple dichotomy belies both the complexity of many human infectious diseases and the myriad potential outcomes of competitive interactions within the host, but it is nonetheless predicted to have a large impact on host health. Is there any evidence that the ‘precise’ diversity of an infection—the number of strains represented by the ‘multiple’ moniker—needs to be considered to understand clinical outcomes?

For HIV infections, the answer seems to be no. A lot can be predicted about clinical outcomes from knowing simply whether an infection was founded by one viral strain or more. Infections founded by more than one strain tend to be more virulent as they yield higher viral loads, which in turn hasten progression to AIDS; in reality, most new infections are founded by single strains [2 and references therein; NB: we use ‘strain’ here for consistency rather than ‘variant’ as favored in that literature]. The preponderance of infections founded by single strains arises in part from the fact that transmission of multiple viral particles is a prerequisite for inoculation with multiple strains. Transmission of many viral particles is particularly likely early on in HIV infections, before viral populations have diversified, and so relatively few strains are available for transmission [2]. Widespread drug treatment should reduce still further the odds of transmission during later stages of infection, and hence inoculations with more than one viral strain [2]. Thus, relying on the simple one-versus-many dichotomy, we may predict synergistic effects of within-host ecology and drug treatment in reducing virulence in HIV infections.

**Figure 1.**
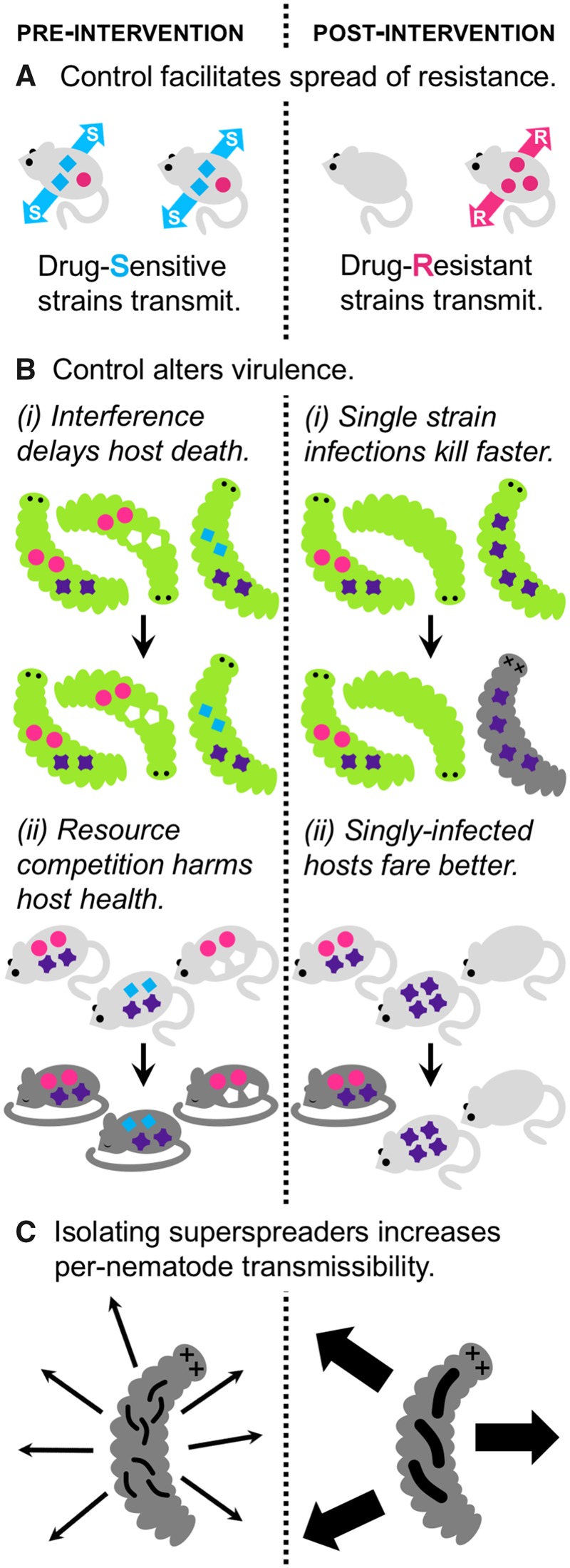
Empirical insights on epidemiological feedbacks driving parasite evolution. (**A**) Reducing transmission could lower multiple infection prevalence and thereby facilitate faster spread of resistance. Competition suppresses the transmission of drug resistance in rodent malaria parasites [3]. (**B**) Reducing transmission decreases multiple infection prevalence, increasing (i) or decreasing (ii) virulence, depending on the mechanism of competition. (i) Entomopathogenic nematodes release mutualistic bacteria (colored shapes) into host caterpillars after invading. Bacteria and nematodes grow separately until host death, when nematodes reacquire bacteria and exit the cadaver. Bacteria strains interfere with one another—delaying host death—by producing bacteriocins [reviewed in 1]. (ii) Coinfecting rodent malaria strains (colored shapes) compete for resources, and diverse infections generate greater anemia [3]. (**C**) Isolating superspreaders generates selection on per-parasite transmissibility*.* Artificial selection for production of many nematodes (black squiggles) emerging from caterpillar cadavers (i.e. superspreading) resulted in smaller nematodes—expected to reduce transmissibility—compared with selection for the production of few nematodes [10]

A similar ‘one-versus-many’ distinction emerges from studying transmission of drug-resistant malaria strains. In untreated rodent malaria infections, drug-resistant strains are competitively suppressed by drug-sensitive competitors and fail to transmit, regardless of whether competitors comprise one or more strains [3]. Unlike our optimistic view for virulence in HIV infections, as control efforts succeed in reducing the prevalence of malaria infections, and coincidentally increasing the frequency of single infections, drug-resistant strains may transmit more efficiently (Fig. 1A). In a further contrast with HIV infections, the number of ‘distinct’ competitors seems crucial for understanding disease severity in this system. In experimental infections, increasing the number of strains elevates the total parasite burden and exacerbates infection-induced anemia [3, and references therein]. Thus, projecting how control efforts will alter virulence in malaria requires understanding the distribution of strains within hosts, resolution that is not required to predict consequences for the spread of drug resistance. Altogether, evidence suggests that comparing single versus multiple infections is sometimes useful (and, indeed, sufficient) for predicting health outcomes, but the patterns vary considerably across and even within systems.

### Do mechanisms underlying competition need to be understood to make public health gains?

There is increasing interest in harnessing competition to achieve particular public health outcomes, for example, delaying or suppressing the emergence of drug resistance [4]. But competition can take many forms, depending on the mechanistic details of within-host interactions (e.g. direct resource competition, immune-mediated apparent competition and interference competition; [1]) and it is not always clear what form (or forms) are at play in any given system. Does this lack of mechanistic understanding preclude exploiting within-host competition for public health gain?

We suggest that the answer to this question is not necessarily. If interventions can be found that mimic the beneficial effects of competition and improve clinical outcomes, then—whatever the underlying mechanisms—those interventions should probably be deployed. However, an understanding of mechanisms is likely to prove crucial for predicting the longer-term consequences of those interventions, and in particular, the parasite evolutionary responses that may erode or bolster their direct effects. For example, intensifying resource competition may select for faster proliferating strains that cause more harm to hosts. In contrast, enhancing interference competition could select for slower-proliferating strains that produce more energetically expensive compounds (e.g. bacteriocins) and cause less harm to hosts [5]. Thus, the same control efforts that reduce transmission, and the prevalence of multiple infections, could have divergent evolutionary consequences for virulence (Fig. 1B), which can only be predicted if mechanisms are known. For public health, an important open challenge is identifying the mechanistic basis for competitive interactions within human hosts, as nearly all current data come from *in vitro* experiments and model disease systems.

The mechanisms underlying competition also determine any priority effects, where an initial infection alters the within-host environment—positively or negatively—for subsequently colonizing strains. An initial infection could make a host more vulnerable to later colonizing strains, for example by exhausting host defenses. Alternately, a combination of resource and immune-mediated apparent competition could reduce the success of parasites that subsequently colonize a host. These priority effects can influence individual health outcomes as well as evolutionary patterns, like the emergence of drug resistance. In projecting the spread of drug resistance, it is useful to recognize that drug-resistant strains are likely to appear at low density in hosts already occupied by drug-sensitive strains. Applied to malaria infections, models show that this numerical disadvantage makes resistant strains more extinction-prone, a disadvantage compounded by priority effects arising from immune-mediated competition [6]. Although the numerical disadvantages (i.e. greater probability of extinction) are likely ubiquitous across systems when resistance arises *de novo* within a host, those risks could be exaggerated or minimized depending on the ecology governing how strains interact. In sum, the bulk of evidence suggests that mechanistic understanding of competition is key to evaluating the long-term efficacy of public health interventions.

### How do within-host interactions scale up to influence epidemiology?

Within-host interactions have the potential to alter critical epidemiological rates such as transmission, virulence and recovery. But are there predictable patterns across, or even within, host-parasite systems? Recovery rates are challenging to quantify, especially for human infections where the timing of inoculation is often unknown. However, times series of human malaria infections—crucially, with known inoculation dates—provide a basis for understanding the drivers of infection length. Childs and Buckee [7] used those data to model transmission and infection duration in single versus coinfections, finding that the addition of a second strain can truncate or extend infection, and may (or may not) make the host more infectious. Outcomes in these cases depended on the timing of the infection and details about host immunity and prior exposure, belying the existence of simple rules for adjusting epidemiological rates in the context of multiple infections.

A related challenge is that when diverse parasites generate similar symptoms, it is difficult to even identify multiple infections, much less estimate their influence on epidemiological processes. For example, Zika, dengue and chikungunya viruses frequently co-circulate and present with similar, dengue-like symptoms, so that infections composed of more than one of these viruses may go undetected [reviewed in 8]. Passive case detection relies on patients presenting at clinics and so cannot reveal whether infections with multiple viruses are more (or less) severe; that determination requires data that are currently lacking on the prevalence of single versus multiple infections among hosts with subclinical infections, i.e. cases where acute symptoms are mild or absent [8]. Importantly, simultaneous infection with two of these viruses can actually inhibit the development of protective immunity against one of them [8], rendering individuals potentially more susceptible to that virus than if they had been exposed sequentially. Thus, predicting the epidemiological consequences of co-circulation, including the impact of coinfection on the progression of future epidemics, depends critically on timing (akin to the priority effects noted earlier). Common principles may become apparent as more data, generated through active surveillance, is brought to bear on the question of individual health outcomes of coinfected hosts and broader patterns of circulation. For now, existing data show that multiple infections alter key epidemiological parameters in hugely varied ways, defying straightforward generalizations.

### What is the evidence for indirect evolutionary effects of disease control?

Direct evolutionary responses to interventions, like drug resistance, are a predictable consequence of disease control, but control efforts may also exert unexpected, indirect evolutionary pressures on parasite traits. Feedbacks between competition and control represent a potentially crucial source of indirect selection; e.g. if control efforts reduce the frequency of coinfections (e.g. Fig. 1A and B), parasites may evolve in response to that altered competitive landscape. Devising experiments to detect those outcomes requires clear theoretical predictions about how parasite traits will evolve, but the complexity and idiosyncratic nature of competitive interactions (as outlined above) makes constructing those models a substantial, ongoing challenge (for an example of such a model, see [6]). Nonetheless, existing theoretical and empirical studies show intriguing possibilities for other unexpected, indirect evolutionary consequences.

Even in the absence of competition, theory demonstrates the potential for indirect selection via epidemiological feedbacks. For example, epidemic expansion can select for earlier transmission from malaria infections and more aggressive parasite proliferation, to the detriment of host health [9]. That outcome arises from the fact that while an epidemic is expanding, most infections are in the early stages. Parasites therefore pay little cost for traits, like aggressive proliferation, that jeopardize transmission late in the course of infection [9 and references therein]. By limiting the frequency of infections in early stages, theory predicts that slowing epidemic expansion should yield public health benefits beyond reducing prevalence [9]. For malaria, those benefits could be even greater if control efforts also reduce the frequency of coinfections thought to be costly to health [Fig. 1B(ii)]. Although multiple sources of indirect selection may align to drive parasite evolution in ways beneficial to public health, other systems may yield more nuanced outcomes; such synergism would not be expected when a reduction in competition hastens host mortality [Fig. 1B(i)]. With further theory across a range of systems, generalities may emerge about when indirect selection is likely to reinforce the public health gains of limiting transmission.

Detecting such long-term, evolutionary consequences of control—especially indirect effects—is a further challenge, but lab studies can nonetheless highlight possible outcomes. For example, artificial evolution experiments suggest that targeting control to superspreaders could have unintended consequences for the evolution of parasite traits underlying transmission. Bashey and Lively [10] artificially selected for entomopathogenic nematodes that produced many or few juveniles after invading their insect hosts. The nematodes selected to produce few juveniles also produced larger ones, expected to be more transmissible to new hosts [10]. Extrapolating from these experimental results, public health interventions that isolate superspreaders (e.g. quarantining hosts with high rates of shedding) could select for increased per-propagule infectivity to compensate for lower shedding rates among the hosts who can still contribute to transmission (Fig. 1C). Depending on the particular tradeoffs at play, such parasite evolution could undercut the long-term benefits of control.

Whether the effect is to enhance or undercut control efforts, theory and experimental data demonstrate convincingly that interventions can impose indirect selection on clinically and epidemiologically relevant parasite traits. A major outstanding challenge is translating this work to human parasites and determining how to detect these subtler evolutionary consequences of intervention efforts. Only then can we evaluate if they represent an important consideration in designing intervention strategies.

## CONCLUSIONS

Within-host competition and public health interventions are both highly potent sources of selection on parasite traits. Failing to account for the potential interactions between these sources of selection can result in over- (or, sometimes, under-) estimating the long-term efficacy of interventions, including resilience in the face of parasite evolution. Comparing across case studies, we find a lack of consistent patterns in how competition alters epidemiology and vice versa, precluding robust, general predictions about parasite evolution. Indeed, current evidence (Fig. 1) suggests a range of potential evolutionary outcomes following control, both positive and negative from a public health perspective. A critical challenge lies in translating theory and experiments in model systems to expectations for evolutionary responses in parasites of global health concern, especially those subject to large-scale control efforts. Guiding principles may emerge from a better understanding of the mechanisms that govern within-host interactions in these cases, narrowing the range of possible evolutionary outcomes. Tracking the knock-on consequences of interventions requires long-term data, ideally obtained through active surveillance efforts that can detect how coinfections contribute to transmission and health burdens. In the meantime, existing data show that the epidemiological feedbacks of altering competition can drive parasite evolution in subtle, but important ways that deserve more attention.
